# Bioprospecting of the agaricomycete *Ganoderma australe* GPC191 as novel source for l-asparaginase production

**DOI:** 10.1038/s41598-021-84949-5

**Published:** 2021-03-18

**Authors:** Meghna Chakraborty, Srividya Shivakumar

**Affiliations:** Department of Microbiology, School of Sciences, Block-1, JAIN (Deemed To-Be University), 18/3, 9th Main Road, 3rd Block, Jayanagar, Bengaluru, 560011 Karnataka India

**Keywords:** Microbiology, Applied microbiology

## Abstract

l-Asparaginase is a therapeutically and industrially-competent enzyme, acting predominantly as an anti-neoplastic and anti-cancerous agent. The existing formulations of prokaryotic l-asparaginase are often toxic and contain l-glutaminase and urease residues, thereby increasing the purification steps. Production of l-glutaminase and urease free l-asparaginase is thus desired. In this research, bioprospecting of isolates from the less explored class Agaricomycetes was undertaken for l-asparaginase production. Plate assay (using phenol red and bromothymol blue dyes) was performed followed by estimation of l-asparaginase, l-glutaminase and urease activities by Nesslerization reaction for all the isolates. The isolate displaying the desired enzyme production was subjected to morphological, molecular identification, and phylogenetic analysis with statistical validation using Jukes-Cantor by Neighbour-joining tree of Maximum Likelihood statistical method. Among the isolates, *Ganoderma australe* GPC191 with significantly high zone index value (5.581 ± 0.045 at 120 h) and enzyme activity (1.57 ± 0.006 U/mL), devoid of l-glutaminase and urease activity was selected. The present study for the first-time reported *G. australe* as the potential source of l-glutaminase and urease-free l-asparaginase and also is one of the few studies contributing to the literature of *G. australe* in India. Hence, it can be postulated that it may find its future application in pharmaceutical and food industries.

## Introduction

l-Asparaginase (l-asparagine amidohydrolase, EC3.5.1.1) for its ability to deaminate l-asparagine (resulting in aspartic acid and ammonia), finds many applications in pharmaceutical and food industries. Due to its role in limiting the availability of l-asparagine in serum, the enzyme is considered to act as an anti-metabolite^1^ and is predominantly known as an anti-neoplastic and anti-cancerous agent^2^. Therapeutically, the enzyme is actively used in management of non-Hodgkin’s lymphoma and acute lymphoblastic leukaemia, among others^2^. Recently, it has also been proposed as a potential molecular target for limiting the pathogenicity of *Mycobacterium tuberculosis*^3,4^ and *Salmonella typhimurium*^5,6^. Further, in active application, l-asparaginase is used in the food industry to mitigate acrylamide in baked and starchy foods^7^. These applications highlight the need for exploring the enzyme for management of cancer and infectious diseases and safe manufacturing of deep fried/baked foods.

Even though distribution of l-asparaginase has been reported widely in plants, animals, and microorganisms, its industrial sources are limited to *Escherichia coli* and *Erwinia chrysanthemi* (in pharmaceutical industry) and *Aspergillus oryzae* and *A. niger* (in food industry)^8^. In clinical applications, the enzyme is delivered either in native form or conjugated with polymers such as polyethylene glycol to develop recombinant form of the enzyme. However, several researchers have reported side effects (namely, development of pancreatitis, aphasia, liver disorders, deep vein thrombosis, hyperglycaemia, and neurological crisis) in case of either forms, especially because of their prokaryotic origin^9^. Although, most of these side effects were alleviated by use of recombinant form, recent reports from certain populations have indicated that the thrombotic and hepatic effects persist even with the recombinant^10,11^.

Other sources of l-asparaginase have not been industrially used till date because of their low catalytic efficiency and stability^12^. These gaps in research are indicative of the need for superior microbial cultures to improve the suitability and production of less clinically-immunogenic l-asparaginase. There is a need to explore the eukaryotic sources, especially fungi, as they can produce high number of extracellular enzymes with more stability, less toxicity, and reduced immune responses^12^. Even though several fungi belonging to the class Eurotiomycetes (*Pencillium* and *Aspergillus*) and Sordariomycetes (*Fusarium*) have been extensively researched for l-asparaginase production, Agaricomycetes fungi (formally known as Basidiomycetes) as a source has been less explored.

Among the wide range of medicinally important agaricomycetous fungi such as *Pleurotus ostreatus*, *Psilocybe semilanceata*, *Ganoderma lingzhi*, *G. lucidium*, *Clitopilus passeckerianus*^13^*, Ganoderma* sp. are widely used with conventional therapy for the management of diseases such as coronary heart disease, chronic bronchitis, leukopenia, and arrhythmia. In case of cancer therapy, besides reduction of their effectiveness due to the resistance from cancer cells, the extensive use of chemical drugs has led to reduction of quality of life of patients in general. *Ganoderma*, often considered as an herbal fungus, is frequently administered in combination with drugs for cancer and has been an effective immunotherapeutic agent towards several types of cancer such as liver, leukaemia, lung, and breast cancer^14^.

However, as per the authors’ knowledge, *Ganoderma* as a source of l-asparaginase has not been reported earlier in existing scientific literature, which is the main find of the present study. With regards to its isolation and research, only one other study from India reported extensively on the isolation of *G. australe* from South India^15^. Hence, the present study attempts to contribute to the less available literature on *G.* *australe* and is the first report on l-asparaginase production by *G. australe*.

## Materials and methods

### Source of agaricomycetes

Healthy basidiomata (fruiting bodies) of mushrooms, growing on dead wood and well-grown trees such as *Delonix regia* and *Eucalyptus globulus*, were collected from various locations in Bengaluru, Kolkata, Tamil Nadu and Pune, India. The samples were collected during the annual monsoon season.

### Specimen treatment and tissue culture

Young and healthy basidiomata were thoroughly pre-washed in sterile distilled water and cleaned with a damp paper towel to remove any dirt and damaged external tissue. The basidiomata were then swabbed with ethanol (70% v/v) to remove any contaminant on the surface^16^. In order to isolate the fungal mycelia in pure form, tissue sections from the inner parts of the fruiting bodies were inoculated on potato dextrose agar (PDA) supplemented with filter-sterilized gentamicin (250 mg/L). Incubation conditions for the growth of fungi were—dark conditions at 28 ± 2 °C for 7–14 days^17^ in a fungal incubator. The pure cultures of each isolate were maintained in PDA plates for further analysis. Basidiomata of the specimens were stored under refrigeration conditions.

### Screening of isolates for l-asparaginase activity

#### Media

The modified Czapek Dox medium (MCD; composition g/L: l-asparagine, 10; KH_2_PO_4,_ 1.5; KCl, 0.5; MgSO_4_.7H_2_O, 0.5; FeSO_4_.7H_2_O, 0.03; CuSO_4_.3H_2_O, 0.03; ZnSO_4_.7H_2_O, 0.05; agar, 20; pH 5.5) was used for determination of l-asparaginase activity. Likewise, for the screening of l-glutaminase and urease activities, the same medium (devoid of l-asparagine) was supplemented with l-glutamine and urea. Phenol red (PR; 0.009% w/v from 2.5% w/v stock solution) and bromothymol blue (BTB; 0.007% w/v from 0.04% w/v stock solution) was supplemented in MCD medium, respectively as pH indicators for the screening assays^18,19^.

#### Qualitative analysis by plate assay method^19^

The sterile MCD agar (1% w/v: l-asparagine, l-glutamine or urea, respectively) was inoculated with the pure cultures of the test organisms using a sterile cork borer (6 mm diameter) and incubated at 28 ± 2 °C for 120 h. Uninoculated media and media without indicator were considered as abiotic and biotic controls, respectively.

l-Asparaginase, l-glutaminase and urease activity was indicated by the change in the colour of the media around the fungal growth (from yellow to pink in case of PR and yellow to blue in case of BTB, due to the shift in media pH from acidic to alkaline). The colony and zone diameters were recorded (in mm) at 72 and 120 h for calculation of zone indices of the potential isolates as per the formula^8^:

$$Zone\, index=\frac{Zone\, diameter}{Colony\, diameter}$$

The isolates demonstrating maximum mean zone indices were considered for further study.

#### Quantitative analysis by Nesslerization reaction^20^

Following the incubation (28 ± 2 °C, 120 h) of the fungal cultures, the fermentation broth (5 mL) was centrifuged (5000×*g*, 5 min, 4 °C), and the quantitative analysis of the enzyme activity was performed with the cell-free supernatant i.e., crude enzyme.

The reaction mixture containing l-asparagine (1.5 mL, 0.04 M, prepared in Tris–HCl buffer of 0.05 M, pH 8.6) and enzyme (0.5 mL) were used to make up the total volume to 2 ml and was considered ‘test’. A ‘blank’ was prepared by adding ammonia-free distilled water (0.5 mL), instead of the enzyme. The ‘test’ and ‘blank’ were incubated (37 °C, 30 min), following which the reaction was stopped by adding trichloroacetic acid (0.5 mL, 1.5 M). The resulting protein precipitate was removed by centrifugation (10,000×*g*, 5 min) and the supernatant was collected. Enzyme activity was determined by performing nesslerization reaction, which was carried on by adding Nessler’s reagent (1 mL) to tubes containing the clear supernatant (0.5 mL) and distilled water (7 mL). All the tubes (including the ‘blank’) were incubated at room temperature for 20 min.

To determine the amount of ammonia liberated as a result of the reaction, a standard curve was initially plotted using ammonium sulphate. For the reactions in the test and the standards, the absorbance was recorded at 480 nm using a UV–vis spectrophotometer (Shimadzu, Kyoto, Japan). Enzyme activity was calculated from the graph by using the formula mentioned below:

$$Enzyme\, activity \left(U/mL\right)= \frac{Amount\, of\, {{NH}_{4}}^{+} \,liberated}{Incubation\, time \times mL\, of\, enzyme\, taken}$$

Further, a unit of enzyme (l-asparaginase/l-glutaminase/urease) was defined as the amount of enzyme which catalysed the formation of 1 µmol of ammonia from substrate (l-asparagine/l-glutamine/urea) per min at 37 °C.

### Morphological characteristics

Morphological identification of the selected isolate was recorded based on the criteria of Ridgeway and Lodge et al.^21,22^ Microscopical features (the hyphal and basidiospore features) of the selected isolate was recorded using compound microscope (magnification 40 ×).

### Molecular identification

The genomic DNA of the selected isolate was extracted using the Fungal Genomic DNA Isolation Kit (Chromous Biotech Pvt. Ltd., India). PCR amplification of 18S rDNA was performed with the use of universal forward and reverse 18S rDNA primers (5′-GTAGTCATATGCTTGTCTC-3′ and 5′-GAAACCTTGTTACGACTT-3′, respectively)^23^. Genetic Analyzer (ABI 3500 XL Applied Biosystems, USA) and Seq Scape software (version 5.2) were used for sequencing the PCR amplicon.

Using BLASTn, the nucleotide sequence of the selected isolate was aligned with the other sequences available in the NCBI database. The nucleotide sequence was submitted to GenBank (NCBI, USA) and provided an accession number.

### Bioinformatics analysis

Sequence alignment was done using the sequences obtained from GenBank and of the selected isolate in the Molecular Evolutionary Genetic Analysis software (MEGA, version 10.1.7). Maximum sequence similarity was allowed by manually adjusting the alignment and introduction of gaps. The best-fit substitution model for the evolutionary analysis was determined using Neighbour-joining tree by Maximum Likelihood statistical method. Gaps/missing data was treated with partial deletion with site coverage cut-off of 95%. For the phylogeny reconstruction, maximum likelihood statistical method was used by using bootstrap method (replications = 500). The phylogenetic relationship between the sequences was also assessed by maximum parsimony for further validation. Tree inference was assessed by Nearest-Neighbour-Interchange method, with the initial tree developed by maximum parsimony^24,25^.

### Statistical analysis

Each experiment was conducted in triplicate and the data was recorded in Microsoft Excel software and has been presented as mean ± standard deviation of the mean (S.D). Statistical analysis was carried out using R software (version 4.0.2). The data were analysed by one-way Analysis of Variance (ANOVA) to determine the significance of the values between the isolates. Post-hoc analysis was performed by using pairwise t-test with Bonferroni P-value adjustment to finalize the potent producer of l-asparaginase. P-value < 0.05 was considered statistically significant.

## Results

Healthy basidiomata of 26 basidiomycetes were isolated from the barks of the trees, cultured and screened for the production of l-asparaginase. Morphologically, the isolates were identified to be from the genera—*Pleurotus*, *Phanerochaete*, *Trametes*, *Lentinula*, *Calocybe*, *Agaricus*, *Flammulina*, *Hypsizygus*, and *Ganoderma*.

### Primary screening analysis of plate assay

Of the 26 isolates, 21 isolates demonstrated growth on MCD medium supplemented with different substrates. Growth on only l-asparagine-supplemented media was observed for 15 isolates; both l-asparaginase and l-glutaminase media supported the growth of 5 isolates; and in all substrate media, growth was observed for 1 isolate. No affinity for either substrates was observed in case of 5 isolates. The observations were same in the case of both the pH indicators (PR and BTB) and time durations (72 and 120 h).

With respect to the zone indices, the mean values of the isolates at 72 and 120 h are represented in Table 1. The mean zone index over different isolates was significantly different with P value < 0.0001. Specifically, GPC191 followed by GPC214 and GPC207 had zone indices > 4 at 120 h. With respect to zone index of each isolate, mean of difference was significantly more for 7 isolates (P value < 0.05) which were finalised for the enzyme activity assessment, namely—GPC191, GPC194, GPC203, GPC206, GPC207, GPC208, and GPC214.

**Table 1:** Zone indices of the organisms at 72 and 120 h.

Isolates	Zone index	
	72 h	120 h
GPC191	3.269 ± 0.003	5.581 ± 0.045
GPC214	2.275 ± 0.366	4.504 ± 0.486
GPC207	2.259 ± 0.191	4.257 ± 0.587
GPC206	2.333 ± 2.309	3.389 ± 2.936
GPC203	1.897 ± 0.092	3.185 ± 0.235
GPC194	1.747 ± 0.362	2.93 ± 0.763
GPC208	1.2 ± 0.872	2.736 ± 1.289
GPC201	1.598 ± 0.363	1.542 ± 0.258
GPC213	1.292 ± 0.475	1.475 ± 0.3
GPC205	1.486 ± 0.7	1.411 ± 0.339
GPC202	2.214 ± 1.002	1.275 ± 0.151
GPC193	1.417 ± 0.144	1.25 ± 0.661
GPC197	1.302 ± 0.054	1.241 ± 0.14
GPC198	0.946 ± 0.437	1.208 ± 0.187
GPC209	1.706 ± 0.202	1.155 ± 0.5
GPC192	0.913 ± 0.654	1.089 ± 0.078
GPC212	2.259 ± 0.449	1.061 ± 0.167
GPC195	1.581 ± 0.5	1.01 ± 0.301
GPC211	0.731 ± 0.668	0.993 ± 0.172
GPC210	0.833 ± 0.289	0.973 ± 0.151
GPC196	1.566 ± 0.503	0.836 ± 0.187

Further, of the 21 isolates, only 5 isolates demonstrated growth and activity in both l-asparaginase and l-glutaminase media at 120 h, namely [isolate (zone index in mean ± SD)]: GPC198 (0.167 ± 0.15), GPC203 (0.67 ± 0.29), GPC207 (0.5), GPC210 (0.32 ± 0.09), and GPC213 (1) and only GPC207 (0.37 ± 0.26). No growth was evidenced on the l-glutaminase and urease plates at 72 h for these isolates.

### Secondary screening analysis by Nesslerization reaction

**Figure 1:**
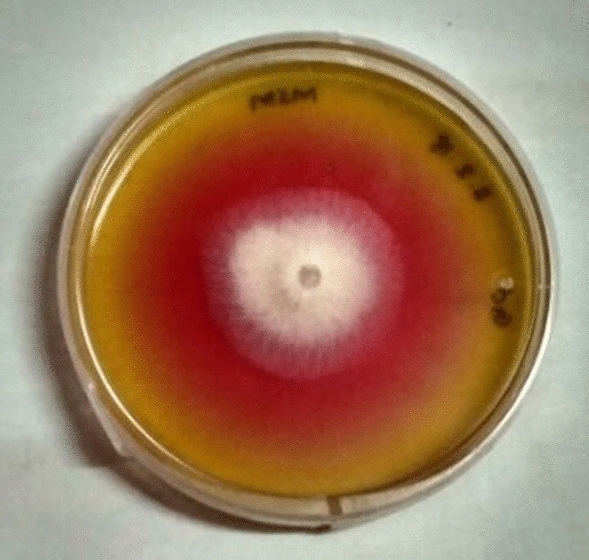
Plate assay showing zone of l-asparagine hydrolysis by GPC191 on modified Czapek Dox medium supplemented with phenol red.

Mean of enzyme activity for all the isolates selected from plate assay analysis is represented in Table 2. Significant difference in the enzyme activity was observed between the isolates (P value < 0.0001). Isolate GPC191 demonstrated highest l-asparaginase activity of 1.57 U/mL. Further, mean of enzyme activity was significantly less for all other isolates as compared to GPC191 (P value < 0.00001). Further, the isolate GPC191 selected for further studies, did not demonstrate any growth or activity in l-glutaminase and urease media. The positive plate assay for l-asparaginase production by the selected isolate GPC191 has been represented in Fig. 1.

**Table 2:** Comparison of enzyme activity over isolates.

Organism	Enzyme activity (U/mL)	P-value
GPC191	1.57 ± 0.006	< 0.0001
GPC194	0.74 ± 0.165	
GPC203	0.47 ± 0.006	
GPC206	0.52 ± 0.006	
GPC207	0.57 ± 0.021	
GPC208	0.35 ± 0.116	
GPC214	0.86 ± 0.14

### Morphological identification of selected isolate

**Figure 2:**
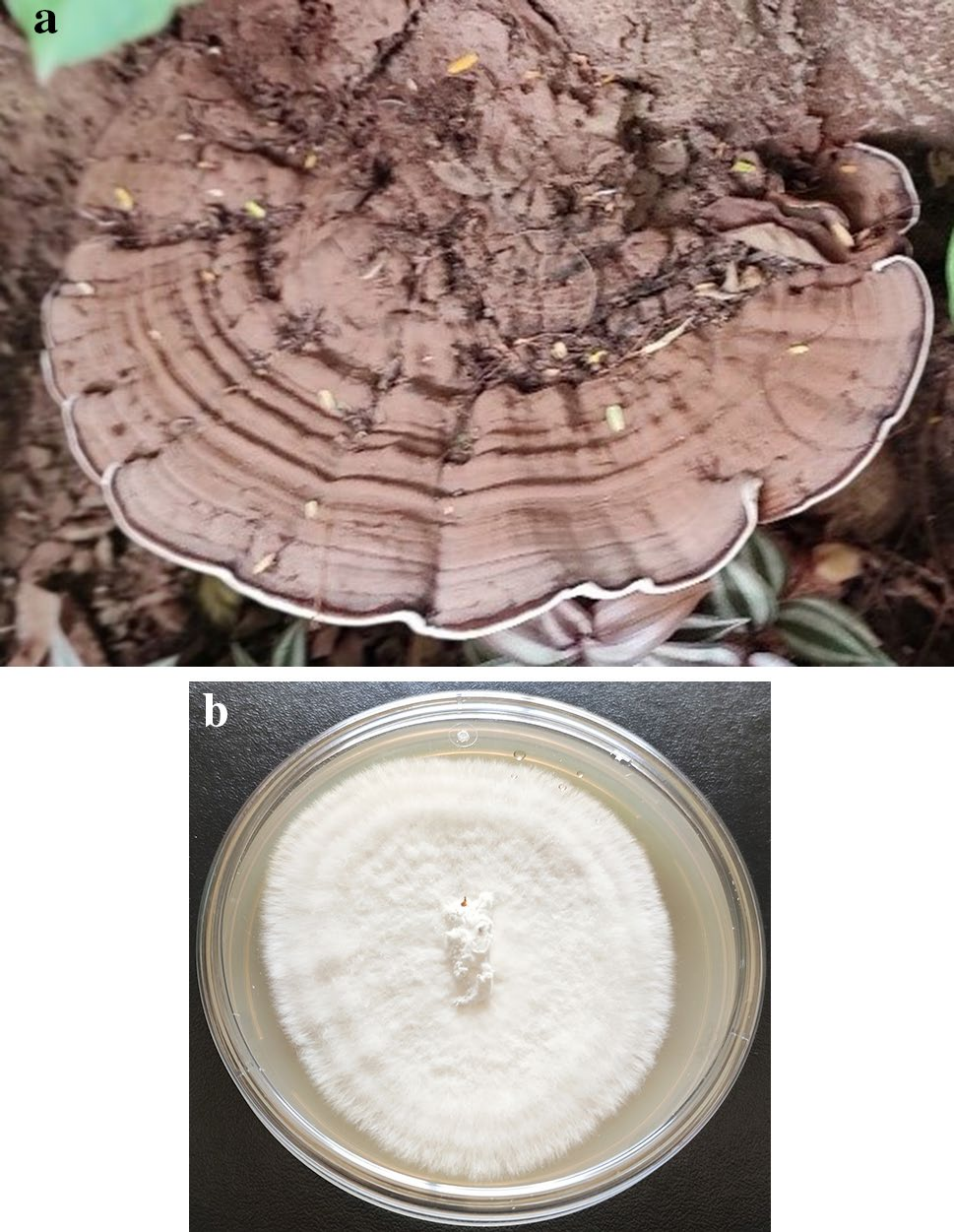
Morphological representation of basidiocarp of GPC191 (**a**) Mature basidiocarp in the field; (**b**) culture plate at 240 h.

Macroscopically, GPC191 isolate showed dimidite, semi-circular, ungulate basidiocarp with prominent fissured, concentric undulations, and rugose surface with reddish grey margin (Fig. 2a). The basidiocarp was 25 cm wide (at broader edge), 10 cm wide (at shorter edge) with approximately 4 cm thickness near the base and 0.5 cm of crust thickness. The pileus was observed to be convex, laccate, hard (throughout), and glaborus with wavy, revolute margin. The stipe was 5 cm wide and 4 cm long, with a thickness of about 3 cm and extended at the base. After 240 h of growth on PDA at 28 ± 2 °C, the culture grew as regular, dense, off-white and velvety mycelia, with scarce aerial hyphae (Fig. 2b).

**Figure 3:**
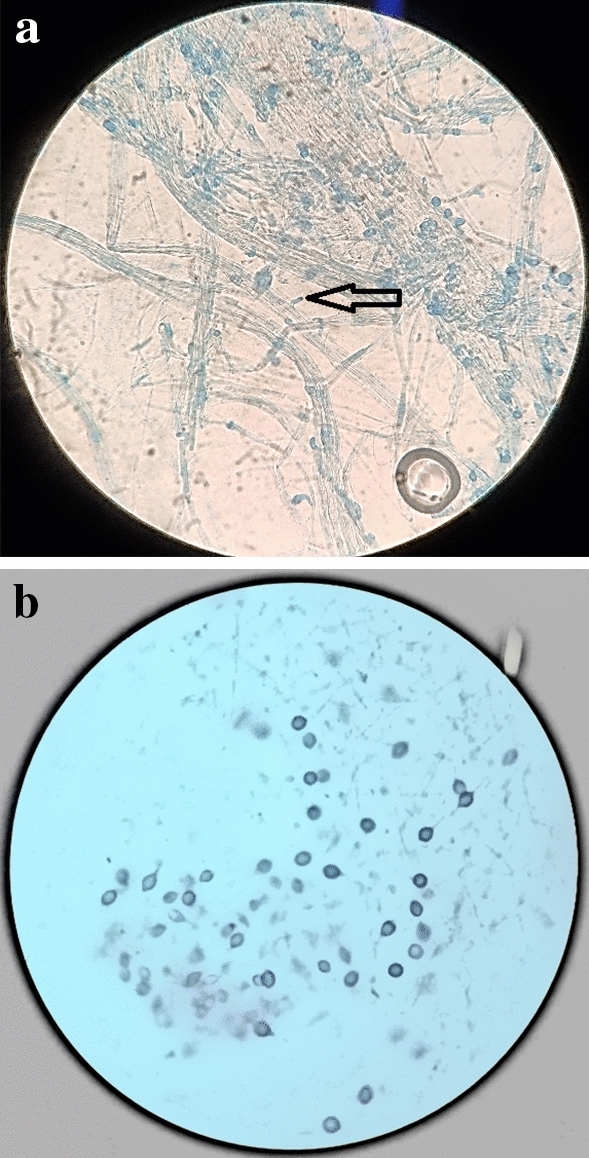
Microscopic images of *G*. *australe* GPC191 (**a**) hyphal system with clamp connections; (**b**) basidiospores. Magnification 40X.

Microscopically, tri-dimitic hyphal system was observed for GPC191 isolate with clamp connections (Fig. 3a). The hyphal system was thin walled and hyaline whereas the basidiospores were ellipsoidal, double-and thick-walled, with distinct tapering observed in some cases and truncated in others (Fig. 3b).

### Molecular identification of GPC191

The 1085 bp 18S rRNA gene sequence of GPC191, obtained after PCR amplification, was compared with registered 18S nucleotide sequences in NCBI database. GPC191 exhibited sequence similarity (99.16%) to that of *G. australe* strain Wu 9302–56. The isolate was, therefore, registered in NCBI as *G. australe* GPC 191 with accession number MN809333.

### Phylogenetic analysis of *G. australe* GPC191

**Figure 4:**
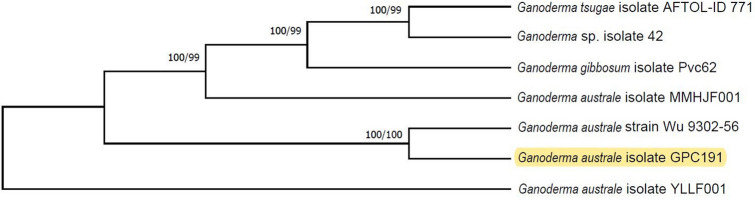
Phylogenetic tree derived from maximum likelihood and maximum parsimony analysis of gene sequences of *G*. *australe* GPC191 (analysed in the study) and of its relatives from GenBank. Bootstrap support values ≥ 70% from 500 replicates of ML and MP analyses are indicated. Phylogenetic tree was prepared using Molecular Evolutionary Genetic Analysis software (MEGA, version 10.1.7, https://megasoftware.net/).

After screening 100 similar sequences from NCBI database, only 7 sequences formed well-supported clades, of *Ganoderma* sp., with high (100/99) bootstrap support values. The best-fit model for evolutionary analysis indicated Jukes-Cantor which was applied to the screened nucleotide dataset to generate the phylogenetic tree. Among the individual clades, *G. australe* GPC 191 formed well-supported clade with type sequence—*G. australe* strain Wu 9302–56 (Fig. 4).

## Discussion

l-Asparaginase, a therapeutically and industrially important enzyme, is widely distributed in plants, animals, and microorganisms. However, among fungal sources, the exploration has been extensively limited to the classes Eurotiomycetes and Sordariomycetes. The class Agaricomycetes has been less explored for their ability to produce l-asparaginase, where only few studies have highlighted *Pleurotus* sp. and *Flammulina* sp. as the sources of the enzyme. Further, studies on bioprospecting the class and identifying the best possible source of l-asparaginase production remains to be reported, which was the focus of the present study.

Isolation of an organism is an important step which assists the researchers to explore the diversity, detect the potential, and identify the best source for production of a desired product by a specific class of organism. Unlike published literature^26–28^ on l-asparaginase production by class Agaricomycetes which concentrated more on screening and production aspect of the enzyme from the pre-selected organisms, the current study focused on isolating and screening a wide variety of mushrooms and narrowing the selection to the best producer of the enzyme. The results of the present study reflected that many Agaricomycetous fungi have the ability of l-asparaginase production.

With respect to screening of the l-asparaginase production, phenol red is the most commonly used indicator for plate assay. However, several studies validate and ensure reproducibility of their results by performing the plate assay with BTB as the asparagine hydrolysing activity of the enzyme is contrasting and distinct in BTB^8,19^. In the case of BTB, enzyme-lysed zone is evidenced as a zone of distinct dark blue colour and the area with the unhydrolyzed substrate remains yellow^29^. Whereas, for phenol red, the contrast and intensity of areas of unhydrolyzed substrate (orange zone) and the enzyme-lysed zone (pink zone) is difficult to observe and differentiate. The present study also used BTB to validate the results obtained from phenol red-supplemented plate assay and ensured reproducibility of the results by comparison of respective plate activity and zone index values.

According to Gulati et al.^18^, the relation between zone index calculated from the plate assay and enzyme activity estimated from the broth is equivalent. In the present study, the zone index values obtained from the l-asparaginase plate assay for the isolates were comparable to results reported by Gulati et al. (*Aspergillus oryzae*, 1.19; *Penicillium nelicum*, 1.02; *Penicillium granulatum*, 0.85)^19^; Doriya and Kumar (C7 strain, 1.57)^18^; and Ashok et al. (*Trichosporon asahii*, 8.0)^8^.

Apart from l-asparaginase activity, the current study also evaluated the production of the l-glutaminase and urease activities by the isolates. Glutaminase activity results in reduction of blood glutamine levels which affects the growth of the normal cells and has consequences such as nerve system dysfunction, pancreatitis, immunological interactions and haemostasis abnormalities, among others. Urease activity leads to elevated levels of ammonia in blood resulting in mild (irritability, vomiting, and headache) and severe (seizures, coma, encephalopathy, and ataxia) symptoms^8,30^. Hence, the presence of glutaminase and urease activities establishes to be detrimental to health and leads to additional downstream processing. It was thus essential, in the present study, to select the isolate that produces l-asparaginase free of these activities. *G.* *australe* GPC191 demonstrated no glutaminase or urease activities, had highest zone index, and enzyme activity values for l-asparaginase.

*G. australe* has nutraceutical and medicinal properties as it is established to be rich in glucans and dietary fibres, and produces bioactive compounds such as lanostane triterpenoids, sterols, and acids (australic, ganoderic, and lucidenic acids)^31–33^. This is, however, the first report on l-asparaginase production by *G.* *australe*. There is only one other report on characterization of l-asparaginase from Agaricomycetes i.e., from *F. velutipes*^26^. But, *F. velutipes* commonly known as the winter mushroom^34^*,* predominantly grows in temperate regions and therefore, is not ubiquitously available. On the other hand, the distribution of *G*. *australe* is worldwide^15^ and thus, the availability of the organism will not be a roadblock for the production of l-asparaginase and in investigating its catalytical properties. For these reasons, it would be advantageous to explore this organism as a potential source of l-asparaginase.

The present study is novel in its approach as it has focused on bioprospecting the class of Agaricomycetes for l-asparaginase production. It also is the first report of *G*. *australe* as the source of therapeutically and industrially-important, l-glutaminase and urease-free l-asparaginase. It also is one of the few studies contributing to the literature of *G*. *australe* in India. Since this organism has proved to be a storehouse of many therapeutic molecules, the large-scale production of l-asparaginase can be hypothesized. Considering the enormous prospect of this enzyme in pharmaceutical and food industries, further studies would be focusing on the nutritional and process parameters that positively influence the enzyme synthesis and specific downstream processing to purify the enzyme.

## Data Availability

The accession number for the *Ganoderma australe* isolate GPC191 registered in NCBI database has been provided in the manuscript. Link for the same is https://www.ncbi.nlm.nih.gov/nuccore/1782692416. The datasets generated during and/or analysed during the current study are provided as table formats; any additional data will be available from the corresponding author on reasonable request.
